# Identification and pathogenicity of a variant porcine epidemic diarrhea virus field strain with reduced virulence

**DOI:** 10.1186/s12985-015-0314-4

**Published:** 2015-06-12

**Authors:** Xiangbin Zhang, Yongfei Pan, Dongdong Wang, Xiaoyan Tian, Yanhua Song, Yongchang Cao

**Affiliations:** State Key Laboratory of Biocontrol, School of Life Sciences, Sun Yat-sen University, 510006 Guangzhou, P R China; Guangdong Wen’s Group Academy, Guangdong Wen’s Foodstuffs Group Co., Ltd., Xinxing, Guangdong China; College of Animal Science, South China Agricultural University, 510642 Guangzhou, China

**Keywords:** Porcine epidemic diarrhea virus, Variant, Reduced virulence

## Abstract

**Background:**

Since 2010, a variant Porcine epidemic diarrhea virus (PEDV), which causes an acute, highly contagious, and devastating viral enteric disease with a high mortality rate in suckling pigs, broke out in China and spread rapidly to neighboring countries, even to the North America. This virus gradually became the main subtype of PEDV worldwide. However, there were no reports of mild pathogenicity of a variant porcine epidemic diarrhea virus in China.

**Findings:**

In 2013, a PEDV-positive sample from a sow with very mild clinical sign was used to inoculate in Vero cells to isolate the virus. This PEDV field strain, designated FL2013 strain, was successfully propagated and genetically characterized. The phylogenetic trees based upon either the complete genome or S gene showed that the FL2013 strain belongs to the genogroup G2b. The S gene of FL2013 has a 7-aa deletion (FEKVHVQ) in the C-terminus comparison with the other G2 PEDV sequences. Further comparative pathology study indicated that the FL2013 strain had reduced virulence to newborn piglets.

**Conclusions:**

A novel variant PEDV strain FL2013 with reduced virulence, as determined by the pathological study, was identified from east China. This strain is closely related to the genogroup- 2 PEDV strains prevalent in the U.S. and China currently, but had a short deletion at the 3′- end of the spike gene.

## Findings

Porcine epidemic diarrhea virus (PEDV) is an enveloped, single-stranded, positive-sense RNA virus in the genus *Alphacoronavirinae* of the family *Coronaviridae*. PED, which is caused by PEDV, is characterized by severe diarrhea, dehydration, and high mortality rates in the affected swine [1]. PEDV variants belonging to genogroup 2 (G2) have been emerging in China in a large-scale outbreak characterized by approximately 80–100 % morbidity rates and high mortality rates among suckling piglets since late 2010 [2]. Highly virulent PEDV strains, phylogenetically related to the G2 Chinese PEDV variants (shared ≥99.5 % nt identity), suddenly emerged in the United States in May 2013 and rapidly spread throughout the country, causing severe economic losses [3, 4]. The second PEDV variant in the US, designated S INDEL PEDV, with insertions and deletions in the N terminal region (S1 subunit) of the spike (S) protein same as the G1 PEDV, was identified subsequently [5]. The S INDEL strains caused reportedly milder disease in the field, indicating that the S gene contains major virulent determinants [6]. The other large S-deletion PEDV strains were also identified, including one Korean field strain with a 204-aa deletion [7], and a US cell-adapted strain with a 197-aa deletion [8]. Whether these S-deletion PEDV strains have distinct pathogenic characteristics, have not been described.

In July of 2013, fecal samples from diarrheal sows, collected in a pig farm in Jiangsu province of east China, were submitted to our lab for routine laboratory diagnostics. PEDV positivity was confirmed by reverse transcription PCR (RT-PCR) according to the method reported previously [2]. One of the PEDV-positive samples, from a sow with very mild clinical sign, was used to inoculate into Vero cells to isolate the virus, as described previously [2]. This PEDV strain, designated FL2013 strain, was successfully passaged and propagated, as characterized by typical PEDV-induced cytopathic effects (CPE), such as cell fusion and syncytia formation (data not shown), which was similar to what we observed for the CHGD-01 strains [2]. The other field PEDV samples collected from pigs with severe clinical sign could not adapt to Vero cells. The complete genomic cDNA of the FL2013 strain was further determined using the 3rd cell culture passaging virus by amplification of twelve regions covering the PEDV genome as described previously [2]. The sequences were assembled and analyzed using DNASTAR program and MEGA6.0 program [9].

**Fig. 1:**
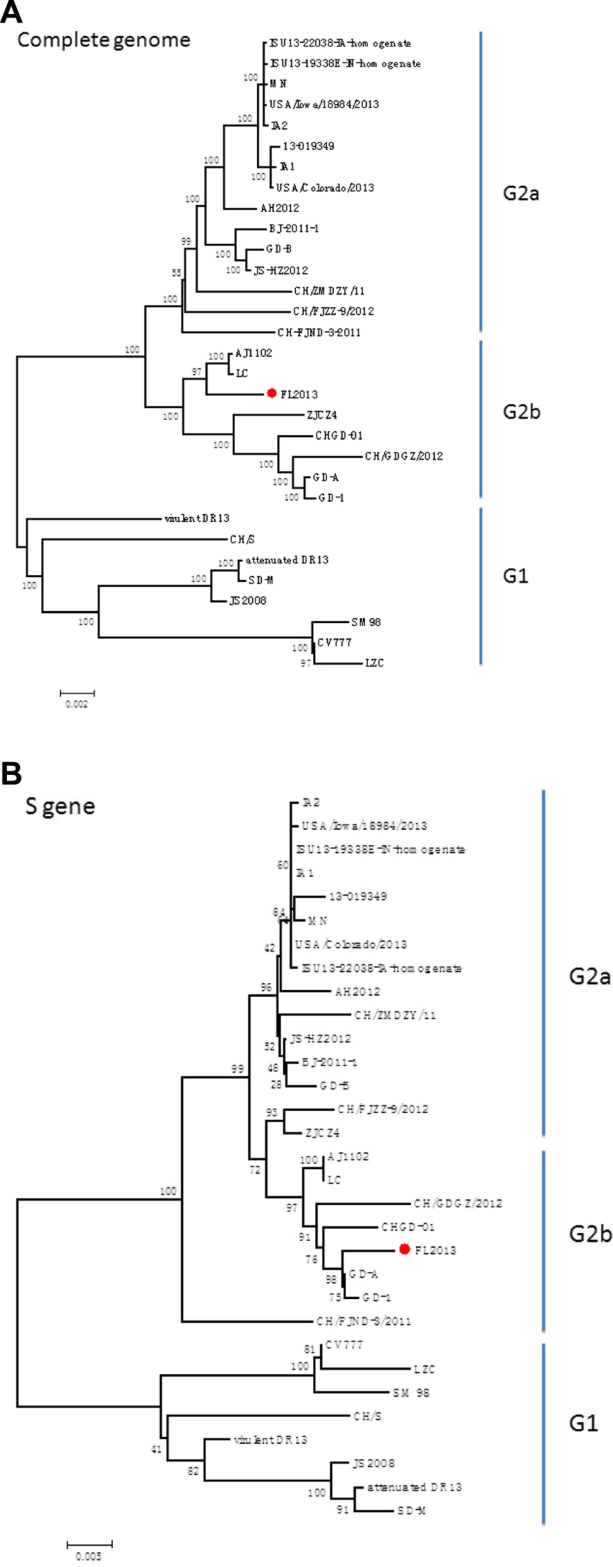
Phylogenetic analyses of representative G1 and G2 PEDV strains based upon nucleotide sequences of the complete genome (**a**) and the S gene (**b**). The trees were constructed by the neighbor-joining method. Bootstrap values are indicated for each node from 1,000 resamplings. The names of the strains, countries of isolation, GenBank accession numbers, and genomic sizes are shown. Red filled circle indicate the PEDV FL2013 sequence in this study

**Fig. 2:**
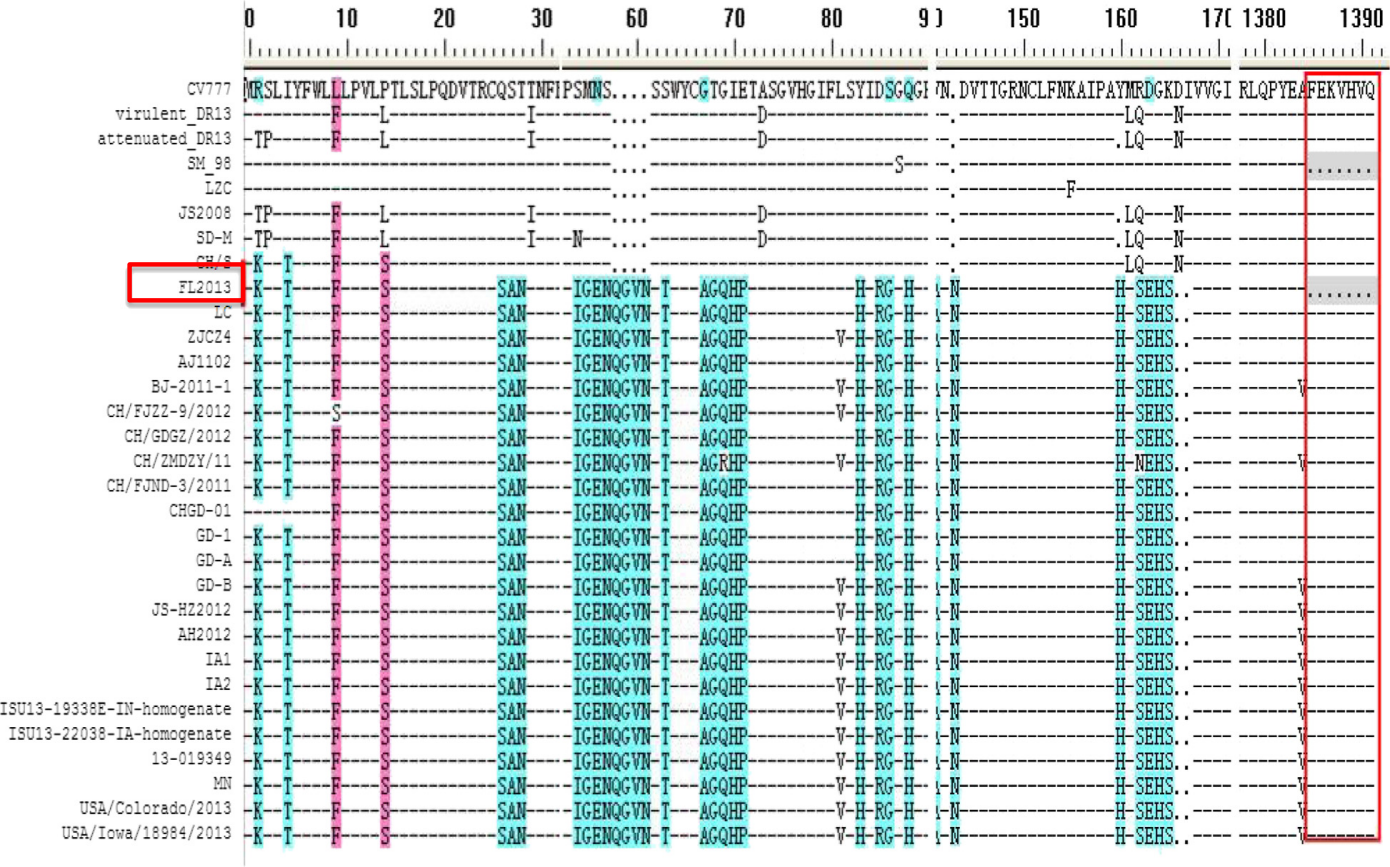
Comparison of the amino acid sequences of selected regions in the S genes among representative G1 and G2 PEDV strains. The strains in G2 had two significant insertions (58QGVN61), 1 (143 N) and a deletion of two aa (DI) between aa positions 167 and 168 at the N-terminus of S gene in comparison with the prototype CV777 strain. Interesting, we found the extreme C-terminus of the FL2013 S gene has a unique 21-nt deletion, leading to a 7-aa deletion (FEKVHVQ) in comparison with the other G2 PEDV sequences

The FL2013 PEDV genomic sequence had the size of 28,044 nt excluding the polyadenosine tail (GenBank accession no. KP765609). Twenty available PEDV sequences with complete genomes were used for multiple alignment and phylogenetic analysis (Table 1). The phylogenetic trees based upon either the complete genome (Fig. 1a) or S gene (Fig. 1b) showed that the FL2013 strain belongs to the genogroup G2b as proposed by Huang et al. [4]. The S gene harbors two significant insertions at aa 58 to 61 (QGVN) and 142 (N), and a deletion of two aa (DI) between aa positions 167 and 168 at the N-terminus in comparison with the prototype CV777 strain (Fig. 2), which is the unique sequence signature of the virulent PEDV strains in the genogroup G2 [2–4]. However, the extreme C-terminus of the FL2013 S gene has a unique 21-nt deletion, leading to a 7-aa deletion (FEKVHVQ) in comparison with the other G2 PEDV sequences (Fig. 2). Interestingly, this unique deletion was also found in a G1 Korean strain SM98 (GenBank accession no. GU937797; Fig. 2). The complete S gene was sequenced in the original fecal sample, the 5th, 10th and 20th cell culture passages, respectively, and no sequence alterations were found, indicating that the 7-aa deletion was naturally present in the PEDV field strain and was stable in cell culture passaging. In addition, we did not find significant mutations on the other structural genes, non-structural genes and untranslated regions (UTR) potentially associated with attenuation.

**Table 1:** Summary of representative PEDV sequences used in this study

Strain	Country	Accession no.	Full length
CV777	Belgium	AF353511	28033 bp
SM98	Korea	GU937797	27994 bp
Attenuated DR13	Korea	JQ023162	27931 bp
Virulent DR13	Korea	JQ023161	28029 bp
LZC	China	EF185992	28042 bp
CH/S	China	JN547228	28026 bp
JS2008	China	KC210146	27954 bp
SD-M	China	JX560761	27953 bp
CH/FJND-3/2011	China	JQ282909	28038 bp
BJ-2011-1	China	JN825712	28038 bp
CHGD-01	China	JX261936	28047 bp
AJ1102	China	JX188454	28044 bp
*FL2013*	China	KP765609	28044 bp
CH/GDGZ/2012	China	KF384500	28035 bp
CH/FJZZ-9/2012	China	KC140102	28038 bp
CH/ZMDZY/11	China	KC196276	28038 bp
GD-1	China	JX647847	28047 bp
ZJCZ4	China	JX524137	28038 bp
LC	China	JX489155	28047 bp
GD-A	China	JX112709	28035 bp
GD-B	China	JX088695	28038 bp
JS-HZ2012	China	KC210147	28037 bp
AH2012	China	KC210145	28039 bp
USA/Colorado/2013	USA	KF272920	28038 bp
USA/Iowa/18984/2013	USA	KF804028	28039 bp
MN	USA	KF468752	28038 bp
ISU13-22038-IA-homogenate	USA	KF650373	28038 bp
ISU13-19338E-IN-homogenate	USA	KF650370	28038 bp
IA2	USA	KF468754	28038 bp
IA1	USA	KF468753	28038 bp
13-019349	USA	KF267450	28038 bp

**Fig. 3:**
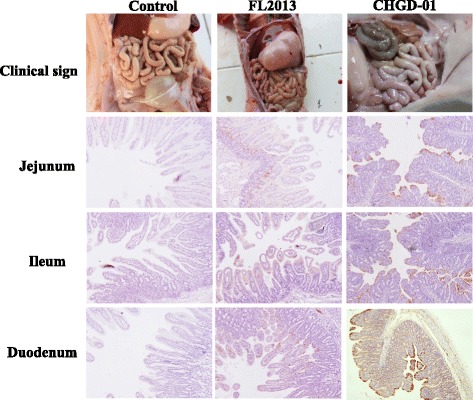
Clinical sign (upper three panels) and immunohistochemical staining for PEDV FL2013 or CHGD-01 strain in tissue section of small intestines. The PEDV antigen stains brown in the cytoplasm of most epithelial cells on the villi (original magnification × 100)

Since the FL2013 strain was isolated from a sow with very mild clinical sign, we were interested to determine its virulence by experimental infection in newborn piglets in comparison with the virulent G2 strain. Thirty-six 3-day-old piglets, negative for PEDV RNA were assigned into three groups with 12 in each. Piglets in each group were housed with their mothers (PEDV RNA and antibody negative) with no artificial supply of colostrum and milk. Group A serves as negative control, whereas piglets in groups B and C were challenged orally with the FL2013 strain and the virulent CHGD-01 strain [2] at 1.0 × 10^5^ 50 % tissue culture infectious doses (TCID_50_)/3 mL, respectively. Two piglets from each group were euthanized and necropsied at 3 days post-inoculation (dpi) for histopathologic examinations and immunohistochemistry staining according to Stevenson et al. [10]. The remaining 10 piglets in each group were examined monitored for clinical signs and recorded to evaluate survival rate for 25 dpi. The result showed that Group-B piglets, together with the negative control group-A piglets did not show clinical sign, and all 10 remaining piglets in each group survived during the observation period (Fig. 3). Group-C piglets had PED signs characterized by acute vomiting and watery diarrhea, and immunohistochemistry staining showed abundant viral antigen in the severely atrophic villi (Fig. 3). In contrast, less PEDV positive staining was found in the intestinal villi of group-B samples, and the villi was only slightly damaged (Fig. 3). 10 of 10 remaining piglets in group C died within 7 dpi. The comparative pathology study indicated that the FL2013 strain had reduced virulence to newborn piglets.

In summary, we identified an apparently attenuated G2 PEDV field strain with 7-aa deletion at the 3′-end of the S gene. Genetic characterizations of various PEDV field strains are of importance for demonstration of PEDV outbreaks and development of efficacy vaccines. Findings from present study reveals that variant PEDV strains circulating in swine-producing areas in China, and provides new molecular epidemiological data for a better understanding of this disease. It is highly possible that the sequence deletions (especially the 7-aa deletion), and other unknown mutations found in the variant strain FL2013 might have contributed to the reduced severity of the clinical disease in the piglets. Whether the changes in the genomic sequence, insertions and deletion, could collectively alter the efficiency of viral replication and RNA synthesis in the FL2013 strain leading to reduced pathogenicity will be investigated in further. More studies will be conducted to test this hypothesis. In addition, the FL2013 isolate may serve as a potential vaccine candidate that could protect the Chinese piglets from the infection caused by the virulent strain of PEDV.
